# Paraneoplastic hyperleucocytosis in a melanoma patient after initiation of ipilimumab and nivolumab combination therapy

**DOI:** 10.1186/s40425-018-0430-y

**Published:** 2018-10-30

**Authors:** Thilo Gambichler, E. Stockfleth, L. Susok

**Affiliations:** Skin Cancer Center, Department of Dermatology, Ruhr-University Bochum, Gudrunstrasse 56, 44791 Bochum, Germany

## Abstract

**Background:**

Paraneoplastic hyperleucocytosis (PH) is sporadically seen in patients with advanced solid tumors.

**Case presentation:**

We report a female patient with disseminated melanoma metastases. Two days after the first dosage of combined immunotherapy using the cytotoxic T lymphocyte antigen-4 (CTLA-4) blocker ipilimumab and the programmed death receptor-1 (PD-1) blocker nivolumab the patient developed asymptomatic hyperleucocytosis (over 120.000 leucocytes per μl) associated with elevated granulocyte colony-stimulating factor blood levels. Hematological and infectious disorders could be ruled out. Although paraneoplastic hyperleucocytosis spontaneously resolved she died from progressive disease about 60 days after start of treatment.

**Conclusions:**

PH is extremely rare in malignant melanoma, however, most patients who developed this complication had preceding immunotherapies such as interleukin-2. The latter observation and the fact that our patient developed PH rapidly after initiation of ipilimumab and nivolumab immunotherapy indicate an immune-mediated mechanism which may trigger PH under unknown circumstances. The development of paraneoplastic hyperleucocytosis indicates a very poor prognosis.

## Background

Paraneoplastic hyperleucocytosis (PH; leucocytes > 100.000/μl) or paraneoplastic leukemoid reaction is sporadically seen in patients with solid tumors, especially advanced lung cancers. PH has extremely infrequently reported in advanced melanoma patients as well.^2-4^ Here we report a particular case of metastatic melanoma-associated PH rapidly developing after the initiation of combined immunotherapy.

## Case presentation

A 72-year old female melanoma patient attended our department in disease stage IV (pT2a, N3c, M1d; AJCC 2017) with a bulky ulcerated tumor mass on the right proximal upper leg, an asymptomatic singular brain metastasis, and further suspected tumor lesions pectoral, iliacal, inguinal and pulmonal. Serum lactate dehydrogenase (LDH) was elevated with 566 U/l (135–214 U/l) and S100B with 0.63 μg/l (< 0.2 μg/l). BRAF, NRAS, and KIT mutation analysis revealed gene wild-types. Based on tumor board recommendation we initiated ipilimumab (3 mg/kg body weight) and nivolumab (1 mg/kg body weight) combination therapy which was granted with accelerated approval by the FDA in 2015 for the treatment of patients with BRAF V600 wild-type, unresectable or metastatic melanoma. Radiotherapy for the brain lesion (stereotactic) and bulky mass on the right upper leg was also planned. Prior to initiation of treatment she had normal blood leucocytes and mild C-reactive protein elevation (CRP).

**Fig. 1:**
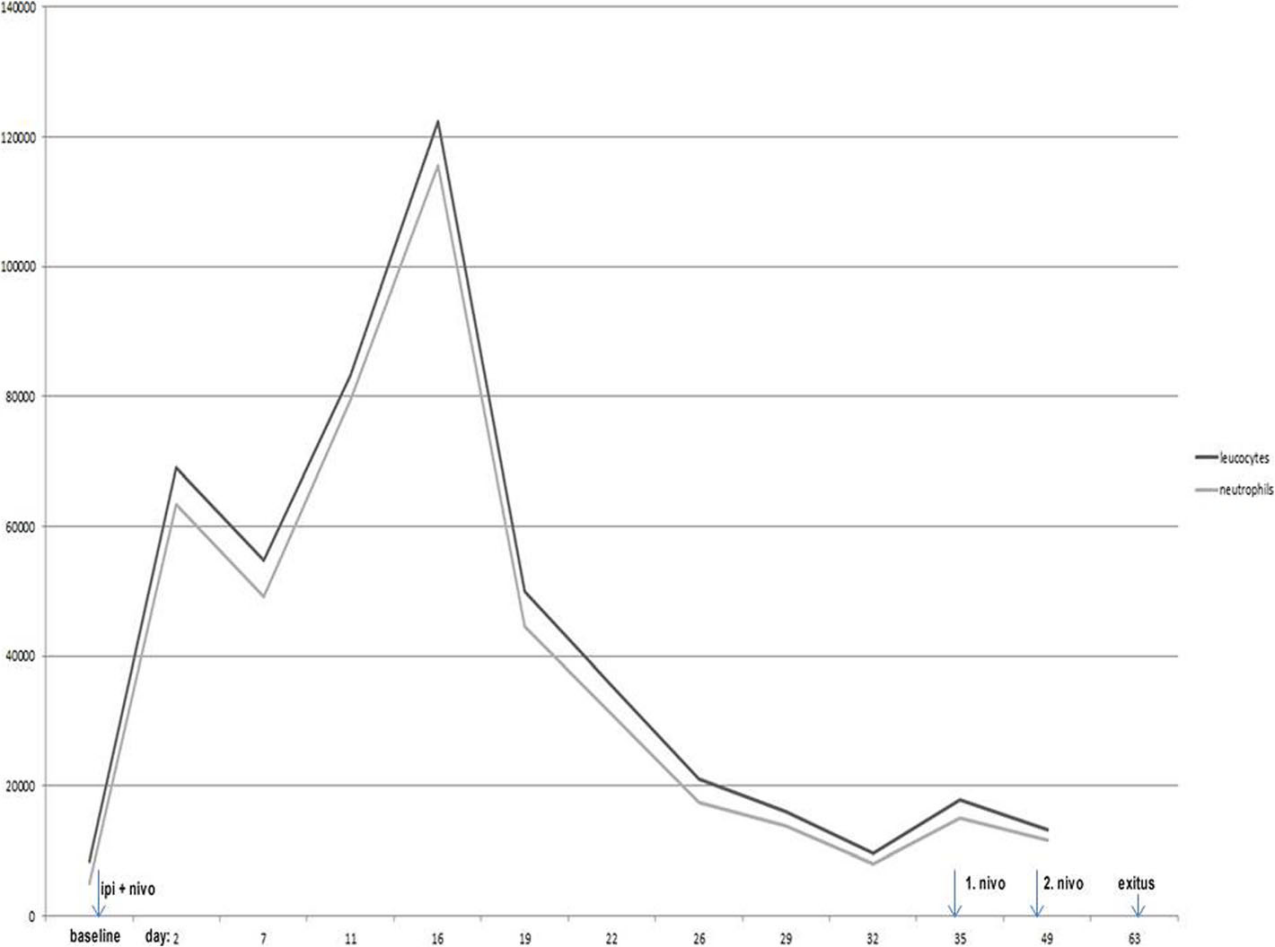
Showing the course of paraneoplastic hyperleucocytosis in a patient with advanced melanoma after initiation of immunotherapy using ipilimumab (ipi) plus nivolumab (nivo)

Two days after initiation of systemic immunotherapy she attended again our department with worsened pain on the right upper leg. Apart from her leg pain she was in good condition without history of chills, fever, weight loss or malaise. However, blood collections revealed a massive leucocytosis (68.970/μl; normal range: 4.600–9.500/μl) with neutrophilia (63.420/μl; normal range: 1.800–7.200/μl). CRP was elevated with 53 mg/l (< 0.5 mg/l). Wound swabs taken from the ulcerated tumor on right upper leg revealed *Staphylococcus aureus*. Hence we administered intravenously 600 clindamycine 3 times daily over 10 days. Blood smears did not reveal signs of leukemia. A bone marrow biopsy was refused by the patient. Procalcitonin was within the normal range. Repeated cultures from blood, urine, and sputum were sterile. Magnetic resonance tomography of the brain and thorax and abdomen computed tomography did not reveal evidence for an infectious focus but demonstrated progress of her tumor condition, including tumor infiltration of musculature on the right upper leg, new pulmonal lesions, and disseminated subcutaneous metastases. LDH and S100B were increased with 588 U/l and 1.27 μg/l, respectively. Granulocyte colony-stimulating factor (G-CSF) was elevated with 33 pg/ml (cut-off: < 21 pg/ml). Granulocyte macrophage-colony-stimulating factor (GM-CSF) was within the normal range. During 2 weeks after initiation of the systemic immunotherapy she developed hyperleucocytosis of 122.360/μl with massive neutrophilia (115.300/μl) as also demonstrated in Fig. 1. Because of her tumor progress and significant spontaneous improvement of her hyperleucocytosis we decided to carry on with nivolumab (fix dosage: 240 mg as approved by the EMA in 2018) monotherapy about 5 weeks after the initiation of the combination immunotherapy. Within the following week her leucocytes even dropped down to 9.600/μl. Since she remained in good condition we continued nivolumab monotherapy and local radiotherapy for the bulky tumor mass on the right leg. Nevertheless, after the second application of nivolumab monotherapy her general condition worsened and she refused further treatment. Two weeks after the last nivolumab infusion she died due to her progressive metastatic disease (Table 1). Interestingly, hyperleucocytosis did not reoccur under her nivolumab monotherapy - her leucocytes were only mildly elevated up to 12.200/μl.

**Table 1:** Clinical course of a female patient with metastatic malignant melanoma (MM) who developed paraneoplastic hyperleucocytosis after initiation of ipilimumab and nivolumab combination therapy

Time	Treatments, interventions	Clinical events
2004, Diagnosis of nodular MM, tumor thickness 1.8 mm, pT2aN0M0, Ib (AJCC 2002)	Sentinel lymph node biopsy negative, Low-dose interferon over 3 weeks, discontinuation due to adverse events	
2004 to 2007	Regular clinical follow-up	
2007 to 2018	Lost on follow-up	
3/2018	Complete work-up including thoracic and abdominal CTs, brain MRT	Admission due to a tumor mass on the right thigh, Asymptomatic singular brain metastasis, and further suspected tumor lesions pectoral, iliacal, inguinal and pulmonal.
4/2018	Tumor board recommendation for ipilimumab and nivolumab combination therapy, radiotherapy for bulky tumor mass, patient refused surgery or cyber knife for brain metastatis	
5/2018	Two days after starting combination therapy Discontinuation of immunotherapy, radiotherapy started	Admission due to a tumor mass on the right thigh, Massive leukocytosis over 60.000/μl
About 2 weeks later	Complete work-up including thoracic and abdominal CTs, brain MRT	Hyperleucocytosis up to 122.360/μl
About 3 weeks later	Continuation of nivolumab monotherapy	Leucocyte counts almost normal
After 2 weeks	nivolumab monotherapy	general condition worsened and she refused further treatment.
After 2 weeks		Patient died due to tumor progression

## Discussion

When a patient with advanced melanoma presents with leucocytosis, in particular with neutrophilia, bacterial infections as well iatrogenic causes such as glucocorticosteroid or hematopoetic growth factor administration have predominantly to be ruled out [1, 2]. However, our patient qualified for PH as she had well-being, did not have fever, did not grow any organism in blood cultures, did not reveal evidence for an infectious focus on extensive imaging, had exorbitant high leucocytosis non-characteristic for infections, and did not show evidence for hematologic malignancies. The latter can also be ruled out since her leucocytosis spontaneously resolved in a short period.

PH is usually due to elevation of G-CSF and/or GM-CSF [1, 3, 4]. Similarly, paraneoplastic hypercalcemia due to parathyroid hormone-related protein production has also been reported previously [5]. Apart from tumor cell-induced G-CSF production, epithelial tumor cells can also express different types of the G-CSF receptors. Paraneoplastic production of growth factors by melanoma cells may thus lead to permanent autocrine stimulation of these tumor cells explaining the uncontrollable tumor progression and poor prognosis of patients with PH [1, 6, 7]. It has also been suggested that G-CSF, secreted by melanoma cells, may suppress T-cell-mediated immune responses against melanoma cells [6]. Moreover, G-CSF can activate RAS/MEK/ERK pathways playing a pivotal role in melanoma pathology [8]. Interestingly, Minowa et al. [4] recently reported a melanoma patient with PH and G-CSF elevation who initially responded to BRAF and MEK inhibition which was paralleled by a marked decrease of leucocytosis [4]. It is conceivable that the decrease of PH might reflect tumor shrinking and thus diminishment of G-CSF producing melanoma cells [4]. Therapeutic G-CSF is widely used in chemotherapy patients, including melanoma patients. Nevertheless, it has been shown that therapeutically given G-CSF might stimulate proliferation of melanoma cells expressing G-CSF receptors. The latter has also been observed in melanoma without PH indicating that the G-CSF expression status might better be investigated before initiating G-CSF therapy in patients with neutropenia [6, 7].

Indeed, a diagnosis of PH is of significance because it has an important prognostic value because the majority of patients with PH tend to have very poor outcome [1]. To our best knowledge, ten prior cases of metastatic melanoma with PH have been reported [1, 3, 4]. Almost all previously reported patients died within the first 3 months after being diagnosed with PH. Importantly, seven of them had pre-treatment with immunotherapy including interleukin 2 (IL-2) [1, 3, 4]. The authors reported, however, that there was no frank temporal relation to IL-2 administration and onset of leucocytosis with neutrophilia [1]. Infact, a well-studied phenomenon of high-dose IL-2 therapy is peripheral lymphocytosis observed in patients receiving treatment for metastatic melanoma [1]. Moreover, it is known that cytokines such IL-1ß, IL-17 and tumor necrosis factor α can induce the production of G-CSF [9].

The association of PH and immunotherapy using CTLA-4 and PD1 blockers has not been reported so far. Notably, our patient developed PH already 2 days after initiation of immunotherapy with ipilimumab and nivolumab. The previously reported coincidence of PH and IL2 therapy in melanoma patients indicate that there might be a pathogenically link between PH and combined immunotherapy in the present case as well [1]. Von Euw et al. [10] concluded from their data that there is a reproducible increase in IL-17-producing cells among activated blood cells after the administration of tremelimumab, another CTLA-4 blocker, suggesting an increase in Th17 cells with CTLA-4 blockade in patients with metastatic melanoma. As mentioned above, IL-17 is an inducer of G-CSF [9]. An increase in IL-17-producing cells among activated blood cells after CTLA-4 blockade might also explain why our patient did not experience PH again under PD1 monotherapy. Although PH may be a rare complication of combined anti-CTLA-4 and anti-PD1 immunotherapy, it is nevertheless of importance given the fact that this treatment modality is increasingly used also in adjuvant settings and other malignancies such as colorectal cancer and renal cell carcinoma. Hence, this treatment complication should not only be aware to dermatologists but also to other specialists treating cancer.

## Conclusions

PH is a rare complication in melanoma patients usually linked to rapid tumor progress and very limited survival. Immunological factors may play a pathogenetic role in the development of PH as also indicated by immunotherapy pre-treatment in the present case. Thus, PH may be considered a very unusual immune-related adverse event of ipilimumab and nivolumab combination therapy. In order to early detect the development of PH one could monitor G-CSF blood levels during the first months of combination therapy.

## Abbreviations

CSF: Granulocyte colony-stimulating factor
CTLA-4: Cytotoxic T lymphocyte antigen-4
G- CRP: C-reactive protein elevation
GM-CSF: Granulocyte macrophage-colony-stimulating factor
LDH: Lactate dehydrogenase
PD-1: Programmed death receptor-1
PH: Paraneoplastic hyperleucocytosis
